# Increasing the use of preventative health services to promote healthy eating, physical activity and weight management: the acceptability and potential effectiveness of a proactive telemarketing approach

**DOI:** 10.1186/1471-2458-12-953

**Published:** 2012-11-07

**Authors:** Luke Wolfenden, John Wiggers, Christine Paul, Megan Freund, Christophe Lecathelinais, Paula Wye, Karen Gillham

**Affiliations:** 1 School of Medicine and Public Health, The University of Newcastle, NSW, Australia; 2 Hunter New England Population Health, Newcastle, NSW, Australia; 3 Hunter Medical Research Institute, , Newcastle, NSW, Australia; 4 School of Psychology, The University of Newcastle, NSW, Australia; 5 NSW Cancer Institute Early Career Research Fellow, The University of Newcastle, C/O Hunter New England Population Health, Locked Bag 10, Wallsend 2287, NSW, Australia

**Keywords:** Health service utilization, Diet, Physical activity, Obesity, Telephone, Translation

## Abstract

**Background:**

Telephone based interventions are effective in promoting health behaviours. The use of telephone based support services to promote healthy eating, activity or weight loss, however, are currently under-utilised. The aim of this study was to assess the acceptability and potential effectiveness of a telemarketing approach in increasing community use of proactive services to encourage healthy eating, physical activity and weight loss.

**Methods:**

The study employed a cross sectional design. Eligible consenting participants completed a 15 minute telephone survey conducted by trained telephone interviewers using computer assisted telephone interviewing technology.

**Results:**

Overall, 87% of participants considered it acceptable for a health service to contact people by telephone to offer assistance to help them lose weight, eat healthily or be more physically active. Among participants with inadequate fruit and vegetable intake, physical activity or who were overweight, 64%, 54% and 61% respectively reported that they would use one or more of the proactive support services offered. Females and those from non -English speaking households who did not eat sufficient serves were significantly more likely to report that they would use support services.

**Conclusions:**

The findings suggest that proactive telemarketing of health services to facilitate healthy eating, physical activity or weight loss is considered highly acceptable and may be effective in encouraging service use by more than half of all adults with these behavioural risks.

## Background

An unhealthy diet, insufficient physical activity, and excessive weight gain are among the primary risk factors of premature morbidity or mortality due to chronic disease [1,2]. While such chronic disease risks are common [3-5], research suggests that there is a strong desire in the community to lead healthier lifestyles. Community assessments of stage of change, for example, suggest that over half of adults are contemplating, preparing or currently trying to improve their diet or activity [6-8]. Similarly population based studies report that between 29-57% of adults are currently trying to lose weight [9-12].

A number of interventions are available to adults in the community to support efforts to improve health behaviours. Systematic reviews have found that intensive behavioural counselling delivered via primary care settings, telephone based support and tailored print materials can improve diet and physical activity and facilitate weight loss [13-17]. Evidence regarding the effectiveness of face to face commercial programs, and those delivered via email or the web remain equivocal [18-22]. Nonetheless, reviews of such interventions have identified the existence of efficacious initiatives [18-22].

Despite interest in healthier lifestyles, few adults use support services to facilitate health behaviour changes. Population surveys indicate that just 3-6% of adults enroll in formal programs [11,23] to facilitate weight loss despite considerable media advertising promoting such programs and less than 35% receive brief dietary, physical activity advice and support from their primary care physician [24,25] despite clinical practice guidelines suggesting physicians should routinely provide preventive care to patients. Similarly research suggests that less than 5% of adults contact telephone services to improve health behaviours [26,27]. For example, despite media promotion, just 4828 adults contacted a free, government funded, state-wide telephone coaching service to improve diet, physical activity and weight loss in the first 18 months of service operation, representing substantially less than 1% of the state’s overweight population [26]. Finally, while the internet is frequently used to search for information regarding physical activity and diet (40-50% of U.S internet users annually) [28], the prevalence of community use of formal web based programs for dietary, physical activity or weight status improvement is unknown. Perhaps unsurprisingly, population surveys report that just 20-30% of adults attempting to lose weight report the recommended practices of eating fewer calories and exercising more [10,12,23].

Television, print, or radio advertisements are often employed to promote the use of preventive health services. While such initiatives can increase service use, they have often been criticised as costly, and typically require adults to take action and solicit support [29]. An alternate method of increasing the initial uptake of support services, is to actively contact and offer support direct to community in an unsolicited manner (i.e. pro-actively recruit). To the authors knowledge, previous studies have not examined the likely impact of such direct tele-marketing of services to encourage healthy eating, physical activity or weight loss. The Puckhet community based cardiovascular disease prevention program, however, utilised unsolicited telephone calls to household-holds to increase use a group based programs to reduce behavioural risk factors for chronic disease [30]. As part of the initiative approximately 25% of all households contacted enrolled in a group program. Similarly, direct contact interventions (in person or via telephone) have been extensively used to increase mammography screening, with a meta-analysis of 25 interventions reportedly increasing attendance from 21% to 46% [31]. Furthermore, a recent study demonstrated that a proactive, cold call, telephone recruitment approach was effective in recruiting 52% of all smokers from randomly selected households in the community to receive smoking cessation support provided by a Quitline, far higher than previous estimates of prevalence of Quitline use in the community (1-7%) [32,33].

The health impact of many support services is also constrained by high rates of program discontinuation, reducing the likelihood of successful behavioural changes [20,22,34,35]. A study of over 60,000 adults enrolling in the Jenny Craig commercial weight loss program, for example, reported that just 22% remained in the program at 6 months [36]. Similarly, trials of web based interventions report substantial reductions in the extent to which users log-on and engage with such programs over time [20]. Proactively providing ongoing intervention contact may reduce the likelihood of program attrition and increase the potential effectiveness of behaviour change strategies. For example, telephone, email or print based interventions are capable of providing tailored intervention support direct to consumers, at a time of their convenience, in a way which does not require users to travel or log on, and which is not reliant on participants to trigger the provision of support [29].

Despite the potential merits of proactive approaches to increase the initial uptake and ongoing use of services promoting a healthy diet, physical activity and healthy weight loss, the feasibility of these strategies require investigation. The aim of this study was to assess the acceptability and potential effectiveness of a telemarketing approach in increasing community use of such services. We also sought to identify possible barriers to, and the demographic predictors of likely use of, such services.

## Methods

The research was approved and the study procedures monitored by the Hunter New England Human Research Ethics Committee and the University of Newcastle Human Research Ethics Committee.

### Study design and sample

A descriptive cross sectional survey was undertaken. The sampling frame for this study was a cohort of participants of an earlier random household telephone health survey who agreed to be contacted and invited to participate in future health research. Randomly selected participants from the cohort were telephoned, assessed for eligibility and invited to participate in this study. To be eligible, participants were required to speak English, be aged over 18 years, reside in New South Wales (NSW), Australia and have at least one of the following health risks: i) a body mass index of ≥25 (based on self reported height and weight) [37]; ii) consume less than 2 serves of fruits or 5 serves of vegetables (based on self reported consumption [37]); iii) participate in less than 150 minutes of physical activity per week (based on self reported activity assessed using the Active Australia survey items) [38].

### Data collection and measures

Eligible consenting participants completed a 15 minute telephone survey conducted by trained telephone interviewers using computer assisted telephone interviewing technology. The surveys items assessed participant demographics, the acceptability of cold calls, and based on their assessed behavioural risks, the likelihood that they would use a telephone support service to improve their diet and physical activity or to lose weight.

#### Participant demographics

During the telephone survey all participants were asked a series of demographic items assessing age, gender, highest educational qualification, household income, and if the participant was Aboriginal or Torres Strait Islander, or spoke a language other than English at home. Items assessing participant demographics were sourced from the ongoing NSW Government Population Health Survey [37].

#### Acceptability of cold calls

All participants were asked if they thought it is acceptable (acceptable, unsure, unacceptable) for a health service to contact people by telephone to offer assistance to help them lose weight, eat healthily or be more physically active.

#### Likely service use

Items to assess likely service use were based on those from previously published studies [29,39]. Participants were read the following statement: ‘I am going to read out a list of possible services. Imagine that, the services were available to you for free, at a time convenient to you’. Participants with a body mass index of ≥25 were asked to report if it was likely (yes or no) that they would use the services to lose weight. Participants who were consuming less than the recommended 2 services of fruit or 5 serves of vegetables were asked if it was likely that they would use the services to help improve their diet, and those not participating in the recommended 150 minutes of physical activity each week were asked if it was likely that they would use the services to increase their physical activity. To reduce participant burden, participants with 2 or more of these self reported health risks were randomly assigned to assessments of likely service use pertaining to one risk only, rather than repeating assessments for each behavioural risk. As such, all participants were divided into three exclusive groups (insufficient fruit and vegetable consumption; insufficient physical activity; or overweight or obese) based on sharing a common risk.

Participants were asked to report likely use of the following four support services previously reported to be amenable to proactive provision on a population basis [29]:

1. Mailed proactive support described to participants as: ‘personalized mailed letters and self-help materials like brochures tailored to your needs to check on your weight loss/healthy eating/physical activity progress and to provide you with information and support’.

2. E-mailed proactive support described to participants as: ‘personalized e-mails tailored to your needs to check on your check on your weight loss/healthy eating/physical activity progress and to provide you with information and support.

3. Proactive telephone support described to the participant as: ‘a we-call-you telephone support and information service for people who want to lose weight/improve their diet/increase the physical activity. This would involve arranging convenient times for the service to call you to support you while you are in the process of attempting to lose weight/improve your diet/increase your physical activity. How often you receive the calls would be up to you’.

4. Interactive voice response (IVR) proactive telephone support described to participants as: ‘an automated pre-recorded phone counselling call tailored to your needs to help you lose weight/improve your diet/increase your physical activity. This would be a “we-call-you” approach; however, the call would be made by a computer and a pre-recorded voice could help you with things like suggesting weight loss/healthy eating/physical activity strategies’.

All participants indicating that it was likely that they would use a service were then asked to report, over a 6 month period, the number of service contacts they would be willing to receive.

#### Barriers to service use

Participants indicating that it was unlikely they would use any of the above services were asked to report the main reason that they would not use each service. Participants were free to nominate any such barriers. Responses to items were then categorised by the research team.

#### Analysis

Data was analysed in SAS version 9.2 statistical software. Descriptive statistics were used to describe the characteristics of the sample, and participant responses to acceptability and service barrier items. Associations between the health risk groups and reports of support service use were evaluated through Chi-Square tests for categorical variables and ANOVA for continuous ones. Multiple logistic regression models, controlling for all demographic variables were used to generate odds ratios and examine demographic associations with reported likely use of any support services by group. All statistical tests were two tailed with an alpha of .05.

## Results

### Sample

Of the 974 randomly selected members of the research cohort, 834 could be contacted. Of these 173 refused study participation and a further 79 were ineligible. The remaining 582 agreed to participate, were allocated to one of three groups based on their health risks and competed the survey. The demographic characteristics of participants in each group are described in Table 1. Participants in the overweight or obese group were significantly more likely to be male and significantly less likely to speak a language other than English at home compared with those in the insufficient fruit and vegetable intake or insufficient physical activity group (p < .01).

**Table 1:** Participant characteristics by group

	**Insufficient fruit and vegetable intake group (n = 324)**	**Insufficient physical activity group (n = 93)**	**Overweight or obese group (n = 165)**	**p**
<50 years of age	43%	41%	33%	0.12
Male	35%	23%	42%	<.01*
Language other than English spoken at home	13%	9%	4%	<.01*
Aboriginal or Torres Strait Islander	1%	1%	3%	.31
University or college education	37%	36%	27%	.07
Annual household income before tax of > $80,000 ^A^	33%	32%	33%	.94

^A^11, 6, and 8 missing responses from the insufficient fruit and vegetable, physical activity and overweight or obese groups respectively were missing due to participant refusal to respond to this item.

#### Acceptability of cold calls

Overall, 87% of participants considered it acceptable for a health service to contact people by telephone to offer assistance to help them lose weight, eat healthily or be more physically active.

#### Likely service use

Among participants with inadequate fruit and vegetable intake, physical activity or who were overweight, 64%, 54% and 61% respectively reported that they would use one or more of the proactive support services. For all groups, mailed proactive support was the most popular support service reported by participants followed by proactive email, telephone and IVR support service (Table 2). There were no significant differences between groups in the prevalence of likely use of these services. Among those who reported that they would be likely to use a service, the number of service contacts participants would be willing to receive was 6 or more across each support service and did not differ significantly between groups.

**Table 2:** Likely use of support services by group

**Support Service**	**Insufficient fruit and vegetable intake group % (95%CI)**	**Insufficient physical activity group % (95%CI)**	**Overweight or obese group % (95%CI)**	**p**
Mail Proactive support	51 (45.5-56.4)	42 (31.9-52.0)	47 (39.0-54.30)	.28
Number of mail contacts (mean, sd)	6 (5.5)	6 (5.9)	7 (6.9)	.13
Email proactive support	42 (36.3-47.1)	30 (20.8-39.5)	38 (30.8-45.6)	.13
Number of Email contacts (mean, sd)	10 (13.4)	10 (9.7)	15 (23.4)	.10
Proactive telephone support	33 (27.6-37.8)	26 (16.9-34.7)	33 (25.6-39.9)	.42
Number of telephone contacts (mean, sd)	10 (9.6)	10 (8.9)	14 (22.0)	.28
Proactive IVR support	11 (7.7-14.5)	12 (5.3-18.4)	10 (5.2-14.2)	.84
Number of IVR contacts (mean, sd)	12 (12.7)	7 (7.3)	15 (24.8)	.41
Any support service	64 (58.6-69.1)	54 (43.6-63.9)	61 (53.1-68.1)	0.20

#### Barriers to service use

The reported barriers to service use among those not likely to use each support service are described in Table 3. The most common barrier for all service types was a lack of interest in changing their health behaviours (26%-39%) followed by a preference to attempt to improve their behaviour without support from proactive mail (16%) email (12%) and telephone (19%) support services and a dislike of the service technology for proactive IVR support (25%). For proactive mail and email services, participants also frequently reported that they would be unlikely to open read or act on information provided (10%-14%) or that such support would not help them (10%-12%).

**Table 3:** Barriers to proactive support service use

	**No desire to change % (95%CI)**	**Prefer to try without help % (95%CI)**	**Service would not help me % (95%CI)**	**Lack of time % (95%CI)**	**Dislike of technology % (95%CI)**	**Not wanting pressure % (95%CI)**	**Prefer face to face % (95%CI)**	**Dislike of phone calls % (95%CI)**	**Unlikely to open, read or act on it % (95%CI)**	**Other % (95%CI)**
Proactive mail	39 (34–45)	16 (12–20)	12 (8–16)	3 (1–5)	1 (0–2)	0 (0–1)	5 (3–8)	n/a	14 (10–18)	10 (6–13)
Proactive Email	25 (21–30)	12 (8–15)	10 (7–14)	3 (1–5)	11 (8–14)	1 (0–2)	3 (2–5)	n/a	10 (7–14)	17 (14–22)
Proactive telephone	38 (33–43)	19 (15–23)	10 (7–13)	8 (5–10)	1 (0–1)	4 (2–6)	6 (4–8)	5 (3–7)	0 (0–1)	9 (6–12)
Proactive IVR	24 (21–28)	11 (8–13)	8 (5–10)	3 (1–4)	25 (22–29)	1 (0–1)	18 (14–21)	3 (2–5)	1 (0–2)	8 (5–10)

#### Demographic associations with service use

Females were significantly more likely to report likely use of a support services among those in the insufficient fruit and vegetable group (OR 1.76; 95% CI:1.05-2.93), and the overweight and obese group (OR 2.02; 95% CI:1.02-4.01) (Table 4). Similarly among those from the insufficient fruit and vegetable group, participants from households where a language other than English is spoken were significantly more likely to report likely use of support services than those that did not (OR 3.09; 95% CI:1.75-7.50). There were no other significant associations between demographic characteristics and reports of service use.

**Table 4:** Associations between demographic characteristics and likely use of any healthy eating, physical activity, or weight loss support services

		**Inadequate fruit and vegetable intake group Adjusted OR**	**p**	**Inadequate physical activity group Adjusted OR**	**p**	**Overweight/obese group Adjusted OR**	**p**
Age	<50	1		1		1	
	50+	0.75 (0.45-1.25)	0.27	0.94 (0.33-2.64)	0.91	0.54 (0.26-1.1)	0.11
Gender	Male	1		1		1	
	Female	1.76 (1.05-2.93)	0.03*	0.45 (0.15-1.38)	0.16	2.02 (1.02-4.01)	0.04*
Language other than English spoken at home	No	1		1		1	
	Yes	3.09 (1.27-7.50)	0.01*	6.28 (0.71-55.21)	0.10	1.16 (0.19-6.79)	0.87
University or college education	No	1		1		1	
	Yes	0.89 (.51-1.54)	0.67	2.25 (0.76-6.69)	0.14	0.64 (0.29-1.39)	0.26
Household income	<$80,000	1		1		1	0.27
	>$80,000	0.99 (0.56-1.78)	0.99	0.44 (0.14-1.42)	0.17	1.54 (0.72-3.29)	

## Discussion

Given the under-utilisation of effective health promotion services to prevent chronic disease [11,23,26], research examining strategies to increase service utilisation is required if the public health benefits of such evidence based services are to be realised. The findings of the study suggest that proactive telemarketing of health services to facilitate healthy eating, physical activity or weight loss is considered highly acceptable and may be effective in encouraging service use by more than half of all adults with these behavioural risks. Furthermore, participants indicated that they would be willing to receive between 6 and 15 intervention support contacts from services over a 6 month period, a surprisingly intensive intervention dose. Collectively such findings indicate that telemarketing of preventative health services may represent an effective strategy in providing evidence based support to adults to reduce their health risks, and has the potential to make an important contribution to reducing the public health burden of chronic disease.

Consistent with community interest in addressing health risks [6-11], overall, interest in support services use was high, particularly for tailored print or email based support. Least popular was telephone support delivered via interactive voice recording, due in part to a dislike of such technology, a finding consistent with previous evaluations of interactive voice response (IVR) technology [29,39]. Nonetheless, the provision of tailored print, mail or IVR health services direct to the community has public health appeal given the capacity for such support to be delivered on a population wide basis at very little cost [14,35,40]. Between one in four and one in five participants indicated an interest in receiving proactive telephone support (via person), far higher than the prevalence of community members with behavioural risks for chronic disease which contact telephone based support services of their own volition [32,33]. The potential effectiveness of proactive telemarketing approaches to recruiting those behaviourally at risk of chronic disease to receive telephone based support is timely, given a need identified in recent systematic reviews for research to facilitate the translation of telephone based diet and physical activity interventions which are now know to be unequivocally efficacious [14,35].

Among those in the insufficient fruit and vegetable, and the overweight or obese groups, women were more likely to report an interest in using support services to improve these health risks. Such findings are consistent with previous literature, [39] and may reflect greater concern regarding their weight, more frequent use of dieting strategies, and greater motivation to improve their health among woman relative to men [9,10]. Adults from households where a non-English language is also spoken were found to be more likely to report interest in at least one service to support dietary (p = .01) but not for physical activity (p=.10) or for weight loss (p = .87). Previous research suggests that self-perceptions of overweight are similar across ethnic groups [41], potentially explaining the lack of a consistent association across each of the health risk groups. Culturally and linguistically diverse groups in the community typically experience a number of unique barriers to accessing health services [42].While participants who could not speak English were excluded from trial participation, the findings are encouraging and suggest that proactively contacting households where a non-English language is also spoken may represent one strategy of connecting culturally and linguistically diverse groups to culturally appropriate prevention services targeting diet. Surprisingly, participants from higher income households or who were University of College educated were no more likely to indicate that they would utilise a healthy eating, physical activity or weight loss support services relative to those less advantaged. Such findings suggest that proactive telemarketing of such health promotion services may be unlikely to further widen the socio-economic disparities in chronic disease [43].

While the findings indicate that proactive telephone contact may be effective in encouraging a substantial proportion of adults to engage in supportive initiatives to improve their diet physical activity or weight status, the advantage of such an approach in increasing the absolute numbers of community members who utilise such a support services relative to the other approaches warrants further investigation. Previous research examining methods to increase use of cancer screening services for example, suggests that small media strategies such as (e.g. video, letters, brochures and flyers) may be effective in increasing service use [44]. Similarly, developing systems to enable referral by clinicians of patients in need of health behaviour improvement may represent an acceptable and effective means of enhancing support service use [45]. Investigating the relative effectiveness and cost effectiveness of such approaches in isolation and in combination should therefore be considered.

An important limitation of the study was its reliance on intended service use. Such reports are likely to represent an over estimate [46]. There are also a number of constraints on the external validity of the study findings. For example, the study recruited participants from an existing Australian research cohort. Such participants may differ systematically from the community in their interest in support service use or perceived acceptability of proactive telemarketing of health services. Furthermore, participants in this study were predominately female, and were from lower income households compared with the New South Wales population [47]. Previous research suggests that women and those from socio-economically advantaged households are more likely to report interest in preventive health services [39]. Future research investigating the unsolicited telemarketing and practice provision of support services in populations more representative of the Australian community and in other jurisdictions internationally is warranted to verify the study findings. Nonetheless, the study provides important formative research for health services interested in increasing the use of evidence based chronic disease prevention services and addresses an under studied yet important issue in public health dissemination [48-50].

## Conclusions

The findings suggest that proactive telemarketing of health services to facilitate healthy eating, physical activity or weight loss is considered highly acceptable and may be effective in encouraging service use by more than half of all adults with these behavioural risks. Given the limited use of existing telephone based support services, further research examining the potential cost effectiveness of employing such techniques in enhancing service use and promoting positive health behaviour changes in the community are warranted.

## Competing interests

The authors declare that they have no competing interests.

## Authors' contributions

JW, KG and LW conceived the study idea. LW lead the drafting of the manuscript and data collection. CL conducted the statistical analysis. All authors contributed to study design, interpretation of analysis, and provided critical comment on manuscript drafts. All authors have read and approve the final version.

## Pre-publication history

The pre-publication history for this paper can be accessed here:

http://www.biomedcentral.com/1471-2458/12/953/prepub
